# Energy Saving Planner Model via Differential Evolutionary Algorithm for Bionic Palletizing Robot

**DOI:** 10.3390/s22197545

**Published:** 2022-10-05

**Authors:** Yi Deng, Tao Zhou, Guojin Zhao, Kuihu Zhu, Zhaixin Xu, Hai Liu

**Affiliations:** 1 School of Electronic and Electrical Engineering, Wuhan Textile University, Wuhan 430200, China; 2 Department of Research and Development, TBEA Co., Ltd., Changji 100089, China; 3 Faculty of Artificial Intelligence in Education, Central China Normal University, Wuhan 430079, China

**Keywords:** palletizing robot, differential evolutionary algorithm, bionic demonstration system, optimization of energy

## Abstract

Energy saving in palletizing robot is a fundamental problem in the field of industrial robots. However, the palletizing robot often suffers from the problems of high energy consumption and lacking flexibility. In this work, we introduce a novel differential evolution algorithm to address the adverse effects caused by the instability of the initial trajectory parameters while reducing the energy. Specially, a simplified analytical model of the palletizing robot is firstly developed. Then, the simplified analytical model and the differential evolutionary algorithm are combined to form a planner with the goal of reducing energy consumption. The energy saving planner optimizes the initial parameters of the trajectories collected by the bionic demonstration system, which in turn enables a reduction in the operating power consumption of the palletizing robot. The major novelty of this article is the use of a differential evolutionary algorithm that can save the energy consumption as well as boosting its flexibility. Comparing with the traditional algorithms, the proposed method can achieve the state-of-the-art performance. Simulated and actual experimental results illustrate that the optimized trajectory parameters can effectively reduce the energy consumption of palletizing robot by 16%.

## 1. Introduction

Palletizing robot is widely used in the manufacturing industry. However, high energy expenditure is a fundamental issue of palletizing robots [1]. At present, the energy consumption problem of traditional palletizing robots is becoming increasingly evident [2]. Enterprises urgently needs new palletizing robots with low energy consumption and high degree of automation [3].

The energy consumption problem caused by palletizing robots should be addressed urgently. Energy recovery and trajectory optimization are the most widely used energy saving methods [4,5]. Numerous factors affect how energy is recovered, so the effect of energy saving is unclear. Conventional palletizing robots usually employ the strategies of best time [6,7] and local optimization [8]. Those approaches can save energy consumption by reducing the running time. However, given the complexity in the workplace, simply reducing the time to optimize the trajectory is often impossible. The effectiveness of the trajectory should be considered when reducing energy consumption, particularly whether the optimized trajectory can achieve the expected handling action.

Modern palletizing robots acquire trajectory parameters by human–computer interaction [9,10,11]. Moreover, different from traditional methods in optimizing objectives [12], modern methods combine sensors to optimize energy sources directly [13,14]. This method can reduce energy consumption and ensure the effectiveness of the trajectory. In this study, a palletizing robot that collects initial trajectory parameters through a bionic demonstration system is called a bionic palletizing robot. Bionic palletizing robot can combine flexibility with high load characteristics. The energy-saving planner can optimize the trajectory of the manipulator and realize the energy optimization of the bionic palletizing robot. The bionic palletizing robot equipped with an energy-saving planner has the advantages of flexibility, high load characteristics and energy saving.

The greatest challenges in energy consumption are the optimal algorithm design [15], model establishment [16,17,18], and parameter identification [19,20,21]. Most researchers have made considerable efforts in the relevant direction [22,23,24]. Zhu et al. selected harmony search algorithm to reasonably allocate the running time of different trajectory points of a robot, which reduces the running time and the corresponding operating energy consumption. However, taking time as the optimization goal easily leads to trajectory deviation and inability to complete the task [25]. In [26], the robot trajectory model constructed by Zhang et al. utilized an input shaping algorithm to process the trajectory parameters under the constraints of joint moments to achieve the smoothing of the trajectory while satisfying the objective optimization. Although the trajectory is smooth, the calculation process is complex, and the calculation speed is slow. Wei et al. introduced the property that neural network can converge quickly to solve the problem of minimizing the energy consumption function and improve the processing speed of trajectory parameters [27]. He et al. first established a multi-factor dynamic model and an energy consumption model of a palletizing robot. Then, they used a genetic algorithm to optimize the model to obtain the trajectory with the minimum energy consumption. Although there exists a certain optimal effective, it is easy to fall into local optimum, resulting in unsatisfactory optimization results [28]. Liu et al. proposed a multi-objective optimization method. The operating trajectory of an industrial robot with the minimum energy consumption can be searched by means of constraints on multiple conditions. This method reduces the energy consumption and ensures the effectiveness of the trajectory, but the impact may occur in operation [29]. The optimization methods of different authors are summarized in Table 1.

This article reposts a novel energy saving planner strategy by introducing the different evolutionary algorithm. The energy saving planner aims at seeking the most optimized the trajectory parameters, which can be collected by the bionic demonstration system. The optimized trajectory should not only reduce energy consumption, but also ensure the completion of work tasks. First, the design of the planner should analyze the structural characteristics of the palletizing robot. The model is established according to the structural characteristics. The connecting rod length, centroid position, and motor torque in the model are taken as system parameters. Second, the optimal objective function is designed. The objective function is the overall power consumption of the manipulator. Energy consumption will be reduced by optimizing the objectives.

In this work, the objective function is proposed to find one optimized parameter group to minimize the energy consumption. To this end, we propose an initial trajectory parameter optimization planner based on the differential evolution algorithm for palletizing robots. The planner optimizes the initial trajectory parameters, which are collected by the bionic demonstration system. The major contributions of this article are summarized as follows:

(i) A novel differential evolution algorithm is introduced, which can obtain the instability of initial trajectory parameters by the bionic demonstration system. The advantage of the proposed method is summed as two aspects, such as ensuring the flexibility of the bionic palletizing robot, and boosting the adverse effects of parameter instability.
(ii) To raise the operating efficiency of the energy-saving planner, the mechanical structure only contains the most important influencing factors. The simplified dynamic model of palletizing robot is combined with differential evolution algorithm to form energy-saving planner.
(iii) Experimental results demonstrate that the proposed energy-saving planner can effectively reduce energy consumption, and combines flexibility, high load characteristics and anti-interference. It can effectively reduce the energy consumption of palletizing robot by 16%.

The rest of this paper is structured as follows. The process of building the energy consumption model of the palletizing robot is presented in Section 2. In Section 3, we first introduce the overall structure of the differential optimization algorithm and the selection of related parameters. Secondly, the overall design of energy-saving planner and the establishment of the objective function are described. Section 4 compares the optimal results of different algorithms and tests the planner under different disturbance conditions. Section 5 summarizes the overall content of the paper.

## 2. Model of the Palletizing Robot

### 2.1. Palletizing Robot

The main work of palletizing robot is responsible for the handling and palletizing of materials in automatic production. The emergence of palletizing robots improves the efficiency of logistics operations in the production process, reduces the labor intensity of staff and ensures the safety of personnel. At the same time, the palletizing robot is a high-power robot in industrial robots. Its working environment is complex, and it is often faced with high speed and heavy load during normal operation. Compared with other industrial robots on automatic production lines, palletizing robots consume a lot of energy due to their special working conditions.

**Figure 1:**
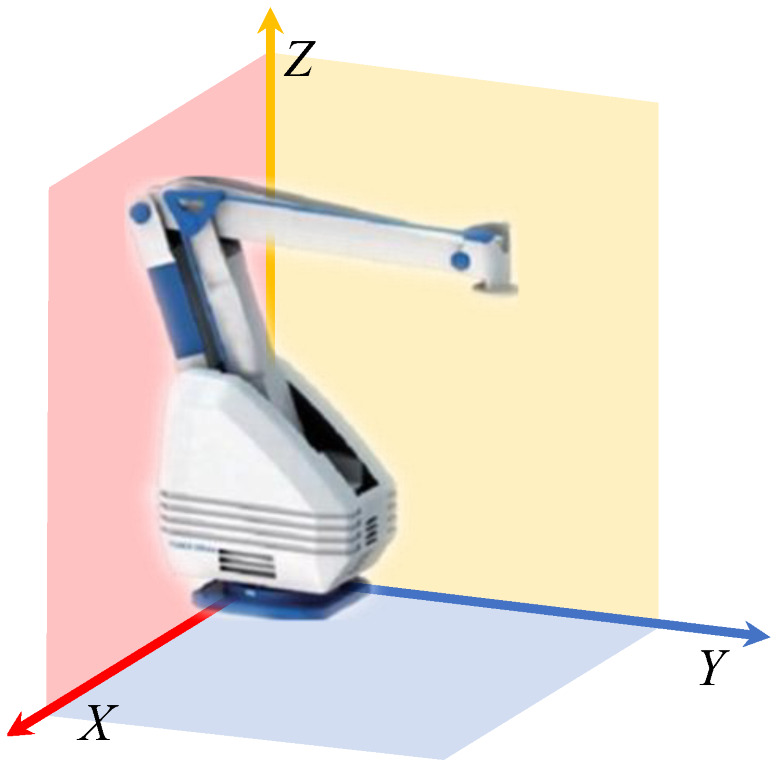
Palletizing robot coordinate system.

The coordinate system of the palletizing robot is shown in Figure 1. The coordinate system takes the rotation center of the bottom fixed base as the origin. The big arm and the small arm are located in the vertical plane composed of *X* axis and *Y* axis. By changing the angle of the driving motor in this plane, the coordinate change of the palletizing robot execution end in a certain range in the vertical plane can be realized. The *x*-axis and *z*-axis constitute the horizontal plane in the coordinate system. Through the rotation of the waist, the coordinate change of the palletizing robot executive end in the horizontal plane is realized. Three servo motors realize the change of the actual position of the palletizing robot in the spatial coordinate system.

**Figure 2:**
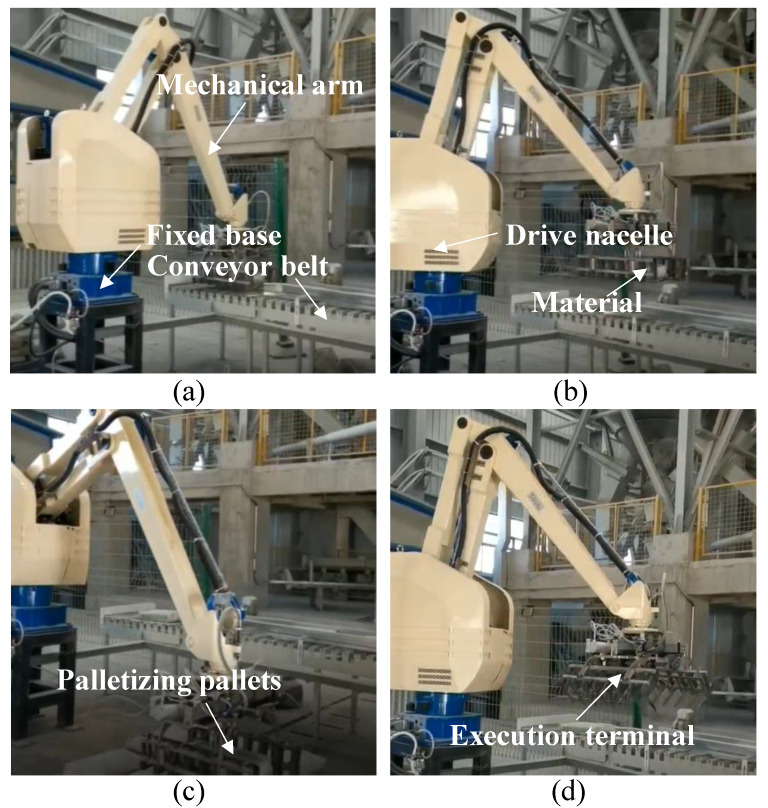
Palletizing robot workflow. (**a**) Grabbing of materials. (**b**) Transport of materials. (**c**) Placement of materials. (**d**) Returning unloaded.

The major structure of palletizing robot includes five parts, such as a fixed base, a rotating waist, a large arm structure, a small arm structure, and a wrist part. The base of palletizing robot is composed of a square fixed disk, which can be utilized to fix the robot arm to the ground or move the mechanism. The waist is driven by servo motors to realize the rotational movement of the robot arm body around the center of the fixed base in space. The large and small arms are driven by two servo motors installed in the drive cabin above the base of the body. The wrist mechanism is driven directly by small servo motors to realize the rotational movement of the end of the robot arm. In Figure 2, the process of palletizing robot in palletizing work is shown. The complete and efficient mechanical structure will be able to effectively cooperate with the bionic demonstration system to realize the palletizing work.

### 2.2. Establishment of the Model

The mechanical mechanism of bionic palletizing robot consists of four joint components. Each component interacts in the operation process, forming a complex mechanical relationship in the whole system. The mechanical relationships of the joints of the mechanical system can be represented by using dynamics modeling. Some of these joints have a high degree of interaction, making it impossible to model each joint separately to study the energy consumption of individual joints. The overall power consumption of the system must be considered as a whole. Palletizing robot modeling methods can refer to the *Springer Handbook of Robotics* is written by Bruno Siciliano. In the study, the Lagrangian equation method is leveraged to model the system dynamics.

The Lagrangian equation of the system is

(1) $$Q_{i}=\frac{d}{dt}\frac{\partial L}{\partial \dot{q_{i}}}-\frac{\partial L}{\partial q_{i}},\;\;\;\;\;\;\;i=1,2,\cdots n,$$

where $Q_{i}$ denotes the generalized moment, L represents the Lagrangian function, $q_{i}$ is the generalized coordinate, $\dot{q_{i}}$ represents the corresponding generalized velocity, and n means the number of the connecting rods. The Lagrangian function *L* is the difference between the kinetic energy *K* and the potential energy *P* of the machine system.

According to the analysis of the mechanical structure of the bionic palletizing robot, the rotation of the wrist of the bionic palletizing robot only determines the operation posture of the end actuator. It has little effect on the overall dynamics of bionic palletizing robot. In kinematic modeling, the influence of the wrist is ignored, and only the dynamics of the waist, shoulder, and elbow are considered.

Through the analysis of the conventional dynamics modeling results. The inertia matrix of the system has nothing to do with the rotation of the waist, but only with the control of the shoulder and elbow joints. Only a few parameters in Coriolis and centrifugal force matrices relate to the rotation of the waist. Thus, the system dynamics can be divided into two parts for analysis. In the first part, the rotational motion of the waist is analyzed as a single-joint system. In the second part, the shoulder and elbow are analyzed as a two-joint system. The operation time of trajectory planning can be effectively reduced by adopting this analysis method.

**Figure 3:**
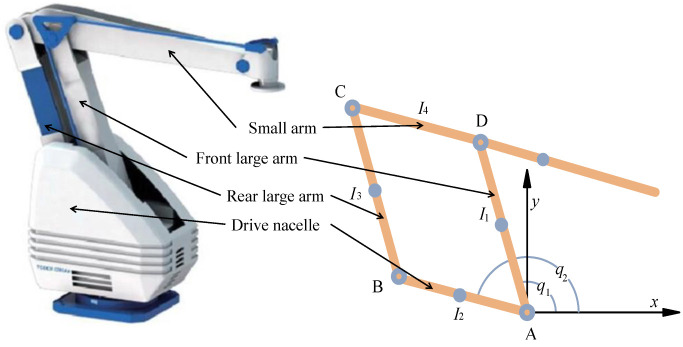
Structural simplification of palletizing robot.

According to the specific structure and simplified factors of bionic palletizing robot, the simplified structures of the shoulder and elbow joints are obtained, as shown in Figure 3. The solid points in Figure 3 are the positions of the center of mass of each rod. $I_{1}$, $I_{2}$, and $I_{3}$ represent the length of the corresponding connecting rod. The length of Rod 4 from Point *C* to Point *D* is replaced by $I_{4}$. The angle between the corresponding connecting rod and the *X*-axis is defined by $q_{1}$ and $q_{2}$.

### 2.3. Shoulder and Elbow Partial Energy Consumption Model

#### 2.3.1. Kinetic Energy Module

Step 1. Calculation of the kinetic energy of the translational part.

First, the centroid coordinates of each connecting rod are obtained. Then, the centroid coordinates of the connecting rod are derived to obtain the centroid velocity.

The centroid velocity from Rod 1 to Rod 4 is

(2) $$V_{c_{i}}=J_{v_{i}}\dot{q},\;\;\;i=1,2,3,4.$$

**Figure 4:**
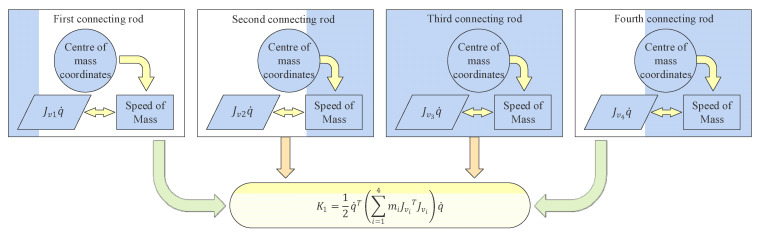
Calculation of translational partial kinetic energy.

From Equation (2), the translational part is derived as follows:

(3) $$K_{1}=\frac{1}{2}{\dot{q}}^{T}\left(\overset{4}{\underset{i=1}{\sum }}m_{i}J_{v_{i}}{}^{T}J_{v_{i}}\right)\dot{q},$$

where $K_{1}$ represents the kinetic energy of the translational part, and $m_{i}$ is the mass of different connecting rods. The detailed calculation process is illustrated in Figure 4.

Step 2. Calculate the kinetic energy of the rotating part.

The following conclusion can be drawn based on the analysis of the simplified structural model of the robotic arm:

(4) $$\omega_{1}=\omega_{3}\;and\;\omega_{2}=\omega_{4},$$

where $\omega_{1}$, $\omega_{2}$, $\omega_{3}$, and $\omega_{4}$ are expressed as the rotation speed of Connecting rod 1 to Connecting rod 4, respectively. The velocity equation is expressed as

(5) $$\left\{\begin{array}{l} \omega_{1}=J_{\omega_{1}}\dot{q}=\left[\begin{array}{ll} 0 & 0\\ 0 & 0\\ 1 & 0 \end{array}\right]\left[\begin{array}{c} {\dot{q}}_{1}\\ {\dot{q}}_{2} \end{array}\right]\\ \omega_{2}=J_{\omega_{2}}\dot{q}=\left[\begin{array}{ll} 0 & 0\\ 0 & 0\\ 0 & 1 \end{array}\right]\left[\begin{array}{c} {\dot{q}}_{1}\\ {\dot{q}}_{2} \end{array}\right] \end{array}\right.$$

The kinetic energy equation of the rotating parts is derived from Equation (5), which can be expressed as follows:

(6) $$K_{2}=\frac{1}{2}{\dot{q}}^{T}\left(\overset{4}{\underset{i=1}{\sum }}I_{i}J_{\omega_{i}}{}^{T}J_{\omega_{i}}\right)\dot{q},$$

By adding Equations (3) and (6), the kinetic energy component of the system is defined by

(7) $$K=K_{1}+K_{2}=\frac{1}{2}{\dot{q}}^{T}D\dot{q},$$

where *D* is expressed as an inertial matrix equation, as follows:

(8) $$D=\overset{4}{\underset{i=1}{\sum }}m_{i}J_{V_{ci}}{}^{T}J_{V_{ci}}+\overset{4}{\underset{i=1}{\sum }}I_{i}J_{\omega_{i}}{}^{T}J_{\omega_{i}}=\overset{4}{\underset{i=1}{\sum }}m_{i}J_{V_{ci}}{}^{T}J_{V_{ci}}+\left[\begin{array}{cc} I_{1}+I_{3} & 0\\ 0 & I_{2}+I_{4} \end{array}\right].$$

After calculation, the element of *D* is obtained as

(9) $$\left\{\begin{array}{l} d_{11}=m_{1}I_{c1}{}^{2}+m_{3}I_{c3}{}^{2}+m_{4}I_{1}{}^{2}+I_{1}+I_{3}\\ d_{22}=m_{2}I_{c2}{}^{2}+m_{3}I_{2}{}^{2}+m_{4}I_{c4}{}^{2}+I_{2}+I_{4}\\ d_{12}=d_{21}=\left(m_{3}I_{3}I_{c3}-m_{4}I_{1}I_{c4}\right)\cos\left(q_{2}-q_{1}\right). \end{array}\right.$$

where $I_{C1}$, $I_{C2}$, $I_{C3}$, and $I_{C4}$ denote the distance between the centroid of Rod 1 and point A, the centroid of Rod 2 and point A, the centroid of Rod 3 and point B, and the centroid of Rod 4 and point D, respectively.

#### 2.3.2. Potential Energy Module

Potential energy is calculated as follows:

(10) $$P=g\overset{4}{\underset{i=1}{\sum }}m_{i}y_{ci},$$

where $m_{i}$ denotes the mass of the different connecting rods, $y_{ci}$ is the coordinate position of the center of the mass of different connecting rods on the Y-axis.

According to the simplified structure of the bionic palletizing robot arm, the expression of the potential energy part can be derived as follows:

(11) $$P=g\;sin\;q_{1}\left(m_{1}I_{c1}+m_{3}I_{c3}+m_{4}I_{1}\right)+g\;sin\;q_{2}\left(m_{2}I_{c2}+m_{3}I_{2}-m_{4}I_{c4}\right).$$

After the derivation of Equation (11), the potential energy part of Lagrange equation can be obtained as follows:

(12) $$g_{1}=\frac{\partial P}{\partial q_{1}}=g\;cos\;q_{1}\left(m_{1}I_{c1}+m_{3}I_{c3}+m_{4}I_{1}\right),$$

(13) $$g_{2}=\frac{\partial P}{\partial q_{2}}=g\;cos\;q_{2}\left(m_{2}I_{c2}+m_{3}I_{2}-m_{4}I_{c4}\right).$$

The structure is made to satisfy the condition $m_{3}I_{3}I_{c3}=m_{4}I_{1}I_{c4}$ when designing the mechanical arm structure of the five-linked rod. Therefore, $d_{12}=d_{21}=0$, and the centrifugal forces inside the system can cancel one another. The system does not produce Coriolis and centripetal forces. The Coriolis and centrifugal force matrices in their Lagrangian dynamics equation will be zero.

The Lagrangian dynamics equation of the shoulder and elbow is simplified as

(14) $$\tau =D\left(q\left(t\right)\right)\ddot{q\left(t\right)+}G\left(q\left(t\right)\right).$$

Equations (9), (12) and (13) are introduced into Equation (14) to obtain the dynamic equations of the shoulder and elbow of the system. The equation is as follows:

(15) $$d_{11}\ddot{q_{1}}+g_{1}=\tau_{1},$$

(16) $$d_{22}\ddot{q_{2}}+g_{2}=\tau_{2}.$$

Equations (15) and (16) show that $\tau_{1}$ separately controls $\ddot{q_{1}}$, and $\tau_{2}$ separately controls $\ddot{q_{2}}$. $d_{11}$ and $d_{22}$ are a constant matrix, independent of the generalized variables of $q_{1}$ and $q_{2}$. The two poles of shoulder and elbow can be independently controlled and will not interfere with each other during operation.

### 2.4. Waist Partial Energy Consumption Model

The end position of the robot arm in the vertical plane is determined by the shoulder and elbow. The rotational motion of the waist joint is mainly responsible for the change of the horizontal spatial position of the robot arm. The mechanical structure of this system can be simplified as a single-joint motion system.

The overall center of mass position of the robot arm is constantly changing when it is working and running. To facilitate a simple calculation, the centroid position of the connecting rod is taken from the center of the maximum arm span of the robot arm to the fixed position. The distance from the center of the maximum arm span of the arm to the center of the fixed base is used to calculate the dynamic equations.

The simplified dynamic equation of the waist system is

(17) $$mr^{2}\ddot{q_{3}}+b\dot{q_{3}}=\tau_{3},$$

where r denotes the distance from the centroid to the rotating center, $q_{3}$ is the rotation angle of the waist, b is the coefficient of viscous friction of the motor, and $\tau_{3}$ represents the torque in the generalized sense.

Combined with the above content, the dynamics model of the three joints of the palletizing robot can be obtained, and the equation is as follows:

(18) $$\left\{\begin{array}{l} d_{11}\ddot{q_{1}}+g_{1}=\tau_{1}\\ d_{22}\ddot{q_{2}}+g_{2}=\tau_{2}\\ mr^{2}\ddot{q_{3}}+b\dot{q_{3}}=\tau_{3}. \end{array}\right.$$

## 3. Energy Optimization Method

According to the analysis of the working characteristics of bionic palletizing robot. A trajectory planner based on the differential evolution algorithm is used to optimize the initial trajectory collected by the bionic demonstration system.

### 3.1. Differential Evolutionary Algorithm

Through the [30] in human and robot collaboration process. When the flexibility of human arm is given to the manipulator, the local optimal solution will appear due to the fluctuation of the human arm when entering the initial trajectory. If genetic algorithm is used, it may fall into local optimum and cannot achieve energy optimization. On the contrary, the differential evolutionary algorithm can well solve this problem.

A differential evolutionary algorithm is a heuristic search algorithm designed according to the natural law of the survival of the fittest. The algorithm has the advantages of good robustness, ease of operation, robustness, and global search capability. At present, it has been applied in different fields. In [31], the derivation steps and algorithm composition of the differential evolution algorithm are introduced in detail. According to the content of the article, we briefly give the main components of the algorithm and the mathematical equation. Differential evolutionary algorithm includes four basic processes: initial population generation, mutation operation, crossover operation, and selection operation.

The initialization of the population is performed to generate the initial population. M individuals of randomness satisfying the requirements are generated in a generalized space of n dimensions. The initialization process is as follows:

(19) $$x_{ij}\left(0\right)=rand_{ij}\left(0,\;1\right)(x_{ij}^{max}-x_{ij}^{min})+x_{ij}^{min},\;\;\;\;i=1,2,3,\cdots ,M;\;\;\;\;j=1,2,3,\cdots ,n,$$

where $rand_{ij}\left(0,\;1\right)$ denotes a random small number between 0 and 1; and $x_{ij}^{max}$ and $x_{ij}^{min}$ represent the upper and lower bounds of the population, respectively.

Then, the variation operation is performed on the initial population by selecting three random individuals from the population, noted as $x_{p1}$, $x_{p2}$, and $x_{p3}$. The variation operation is rewritten as

(20) $$h_{ij}\left(t+1\right)=x_{p1j}\left(t\right)+F\left(x_{p2j}\left(t\right)-x_{p3j}\left(t\right)\right),$$

where $x_{p2j}\left(t\right)-x_{p3j}\left(t\right)$ denotes the differentiation vector; F represents the variation factor; and p1, p2, and p3 denote different integers randomly selected to represent the position of the individual in the population. This part of the operation is the core of the differential evolution algorithm.

Thereafter, the crossover operation is performed. Crossover operations aim to increase diversity in the population. The crossover operation is rewritten as

(21) $$v_{ij}\left(t+1\right)=\left\{\begin{array}{l} h_{ij}\left(t+1\right),\;randl_{ij}\leq CR\\ x_{ij}\left(t\right),\;randl_{ij}>CR, \end{array}\right.$$

where $rand\;l_{ij}$ represents the random decimal between 0 and 1; and *CR* is the crossover probability, which takes values between 0 and 1.

Finally, there exists the selection operation, which aims to judge whether the population after the mutation and crossover operations can become a new member of the next generation.

The previous generation is compared with the new generation through the evaluation function. The results of the comparison are used for selection. The selection process of comparison can be expressed as

(22) $$x_{i}\left(t+1\right)=\left\{\begin{array}{l} v_{ij}\left(t+1\right),\;f\left(v_{i1}\left(t+1\right),\cdots ,v_{in}\left(t+1\right)\right)<f\left(x_{i1}\left(t\right),\cdots ,x_{in}\left(t\right)\right)\\ x_{ij}\left(t\right),\;\;\;\;\;\;\;\;\;f\left(v_{i1}\left(t+1\right),\cdots ,v_{in}\left(t+1\right)\right)\geq f\left(x_{i1}\left(t\right),\cdots ,x_{in}\left(t\right)\right). \end{array}\right.$$

**Figure 5:**
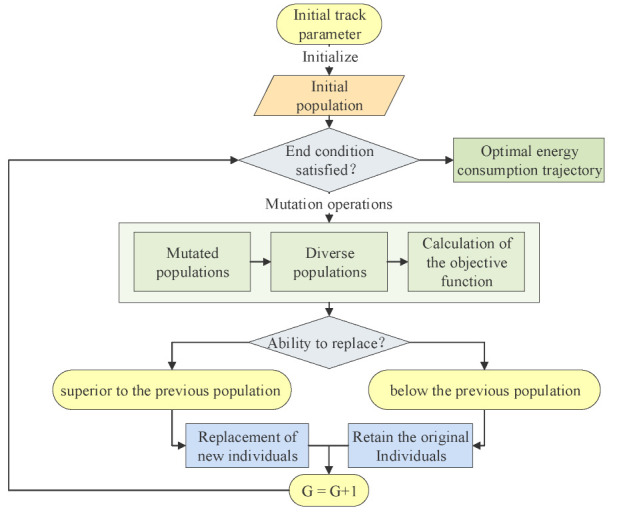
Initial track parameter variation, crossover, and selection form optimal trajectory.

After repeatedly performing the variation-to-selection operation process and upon reaching the maximum number of iterations *G*, the differential evolution algorithm is completed. The above steps are shown in Figure 5.

The selection of some parameters in the differential evolution algorithm is worth noting. The main parameters are the scaling factor *F*, the crossover factor *CR*, the maximum number of iterations *G*, and the population size *M*. For different optimization objectives, the parameter settings of the evolutionary algorithm are also changed according to their characteristics.

### 3.2. Initial Trajectory Parameters

#### 3.2.1. Acquisition of Trajectory Parameters

The trajectory parameters are collected by the bionic demonstration system. Bionic demonstration system is a control method for the height coordination between human arm and mechanical arm. The system gives the human arm flexibility to the palletizing robot manipulator. This approach combines flexibility with payload capabilities, allowing handling tasks to be performed more efficiently and safely. Bionic demonstration system uses the trajectory sensor system installed on the human arm to collect the relative spatial position of the human arm actuator and program the palletizing robot.

The trajectory sensor system consists of a shoulder angle sensor, an elbow angle sensor, a wrist angle sensor, and a gyroscope mounted on the human arm. The sensors in different parts synchronize the collected data to the upper machine in real time. The upper computer analyzes and synthesizes the trajectory of the arm executive terminal. The processed information is transmitted to the lower computer of the palletizing robot to realize the preliminary acquisition of the bionic trajectory parameters of the palletizing robot.

#### 3.2.2. Bionic Trajectory Parameter

The trajectory of the pendulum form is selected as the trajectory parameter collected by the bionic demonstration. The energy-optimized trajectory design is performed for it. The expression of the pendulum trajectory is defined by

(23) $$\theta_{r}=\left(\theta_{ep}-\theta_{init}\right)\left[\frac{t}{T_{Z}}-\frac{1}{2\pi }\sin\left(\frac{2\pi t}{T_{Z}}\right)\right]+\theta_{init},$$

where $T_{Z}$ is the period of the pendulum, $\theta_{init}$ represents the initial angle of the joint, and $\theta_{ep}$ denotes the target angle of the joint.

Given that the differential evolution algorithm is a discrete evolution algorithm, the continuous pendulum motion trajectory needs to be initially discretized. The sampling time interval is taken as $T_{Z}/2n$, and the discrete reference trajectory that can be optimized by the differential evolution algorithm is then derived. The trajectory is given by

(24) $${\bar{\theta }}_{r}=\left[{\bar{\theta }}_{r_{1}},{\bar{\theta }}_{r_{2}}\ldots \ldots {\bar{\theta }}_{r_{2n-1}},{\bar{\theta }}_{r_{2n}}\right],$$

where ${\bar{\theta }}_{r_{j}}$ represents the sampled value of the discrete pendulum motion trajectory at $t=\left(j/2n\right)T_{Z}$.

Define $\Delta {\bar{\theta }}_{J}\left(k\right)$ as the deviation between the reference trajectory. The joint angles are optimized by the differential evolution algorithm. Then, the optimized correction angle is obtained as

(25) $${\bar{\theta }}_{opj}\left(k\right)=\theta_{r_{j}}+\Delta {\bar{\theta }}_{j}\left(k\right),$$

where ${\bar{\theta }}_{opj}\left(k\right)$ represents the optimized joint angle after k iterations of the differential evolution algorithm. k represents the kth iteration of the differential evolution algorithm.

### 3.3. Design of Energy Saving Planner

Assuming that the time that the bionic palletizing robot can allow to reach the required steady state is 3 s, the energy consumption objective function for the *i*th robot arm is expressed as

(26) $$W_{i}=\omega_{i}\int_{0}^{3}\left|\tau_{i}\dot{q_{i}}\right|dt+\left(1-\omega_{i}\right)\int_{0}^{3}\left|q_{op_{i}}\left(t_{j}\right)-q_{r_{i}}\left(t_{j}\right)\right|dt,$$

where $W_{i}$ represents the objective function of the corresponding manipulator, $\tau_{i}$ denotes the generalized moment in the dynamics model, and $q_{op_{i}}\left(t_{j}\right)-q_{r_{i}}\left(t_{j}\right)$ is the distance between the actual tracking trajectory and the optimized one.

According to the mechanical structure constituted by the shoulder and elbow of the bionic palletizing robot arm, the objective function of its system is given by

(27) $$J=\overset{n}{\underset{i=1}{\sum }}J_{i},\;\;\;n=3.$$

After the objective function is constructed, a differential evolution algorithm is used to optimize the reference trajectory. In this way, a trajectory with minimum energy consumption is obtained.

The main parameters of the differential evolution algorithm are set, namely, the maximum number of iterations *G*, the population size *M*, the crossover factor *CR*, and the variation factor *F*. The above parameters should be chosen reasonably according to the objective function to be optimized. After the optimization of the differential evolution algorithm, the discrete optimal trajectory of each robotic arm corner joint can be obtained.

The optimized discrete trajectory needs to be continuous as the optimal tracking trajectory of the robot arm joint angle of this bionic palletizing robot. The discrete optimal trajectory is continuous by the method of the third spline interpolation. The continuous function is obtained after the interpolation optimization is used as the ideal trajectory of the joint angle of the robot arm. To simplify the calculation of tracking the ideal trajectory, the proportional-derivative control algorithm is used to track the ideal trajectory under the condition of neglecting gravity and applied disturbance.

### 3.4. Optimization Process of Running Trajectory

**Figure 6:**
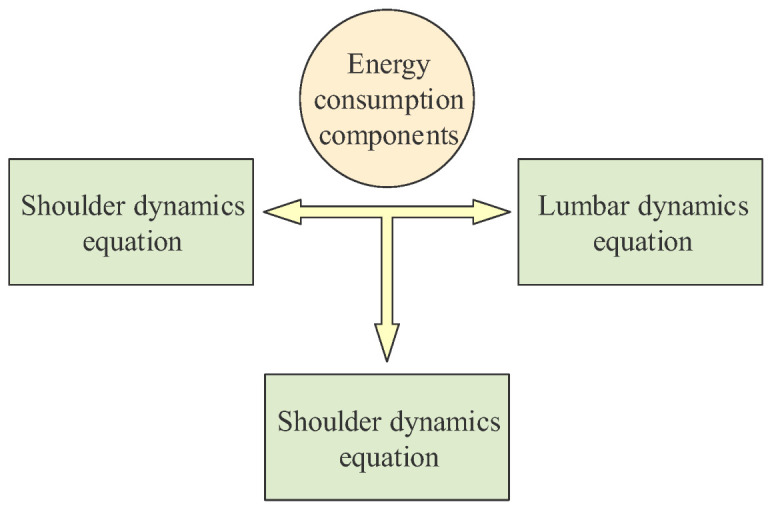
Energy consumption components.

The optimization process is mainly divided into two parts: model building and parameter selection. The robot arm of the bionic palletizing robot is used as the controlled object, and the dynamics model of this system has been established in the previous sections. The approximate structure is shown in Figure 6.

The research objective of this paper is a SAR-50 vertical multi-joint palletizing robot. The robot has a standard four-axis degree of freedom, an effective load of 50 KG, a repetitive positioning accuracy of ±0.5mm, and a body weight of 450 KG (including the drive motor and body shell). This type of palletizing robot has the advantages of large load, good stability, energy saving and environmental protection. The specific parameters are shown in Table 2.

After the model is established, the parameters of the algorithm need to be selected, and the selection needs to be tried continuously during the operation. The larger the value of *M* is, the stronger the initialized population diversity will be and the more likely the optimal solution will be obtained; however, the optimization time will be longer. According to actual requirements, the number of samples selected is 60. The variation factor is used to change the diversity and convergence of the initialized population, and the range of values is usually between 0 and 2. When the value is small, the variation between populations decreases, leading to premature convergence without jumping out of local extremes in the evolutionary process. When the value taken is larger, jumping out of the local extremes is easier, but at the same time, the speed of convergence will decrease. After testing, the variation factor *F* is set as 0.9.

The crossover factor is used to control the participation of individuals from different populations in the crossover operation and balance the global and local search abilities. Values usually range from 0 to 1. The smaller the value of the crossover factor is, the smaller the diversity of the populations will be, leading to premature convergence and inaccurate optimization results. The larger the value of the crossover factor is, the faster the convergence rate will be reduced. After several tests, the suitable crossover factor *CR* is 0.9.

**Figure 7:**
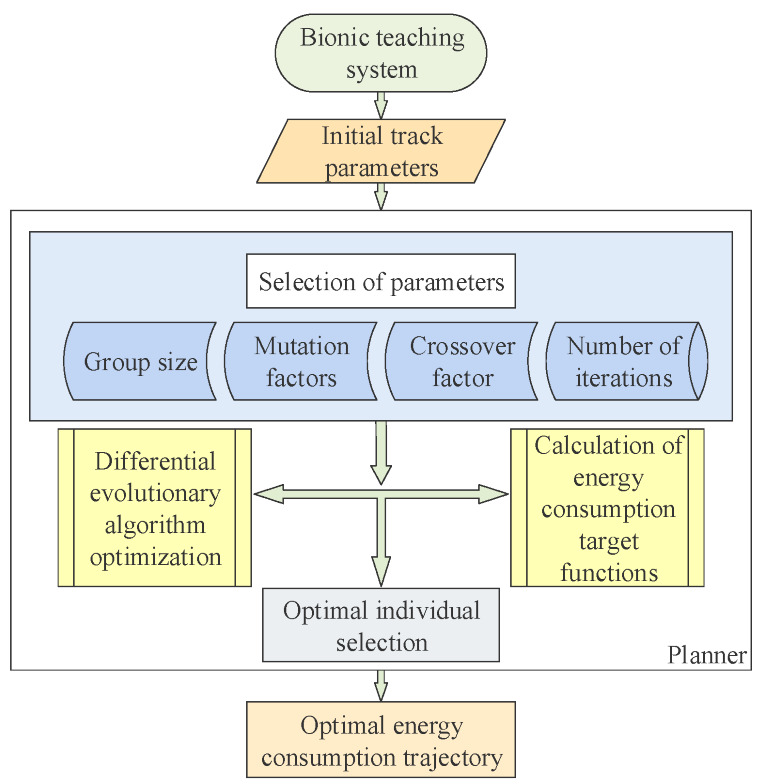
Process of energy saving planner reducing energy consumption of trajectory operation.

The larger the value of the maximum iteration number *G*, the more accurate the optimization result will be. However, with it, the time of optimization calculation will also increase. Therefore, the choice should be made according to the actual situation of optimization. According to the test of the energy consumption model, the maximum number of iterations is set to 20. Through the above process, the initial trajectory parameters are optimized by the energy-saving planner to the optimal energy consumption trajectory. In Figure 7, the optimization process is summarized. First, the main parameters of the planner are set, combined with the actual energy consumption model and the characteristics of the above parameters. Second, the initial trajectory parameters are optimized for energy reduction. Finally, the trajectory of palletizing robot with optimal energy consumption is obtained.

## 4. Experimental Result and Discussion

Optimization effects of planners based on different algorithms are compared. The optimization effect of the planner based on differential evolution algorithm on trajectory and energy is analyzed. Under different interference environments, the optimal effect of the planner based on the differential evolution algorithm is tested.

### 4.1. Comparison of Track Optimization Results

In some literature, a Fourier approximation was used to construct the energy consumption model of a palletizing robot. The energy of the drive system was optimized by genetic algorithm with good results. In this study, the genetic algorithm is used for comparison with the differential evolution algorithm.

**Figure 8:**
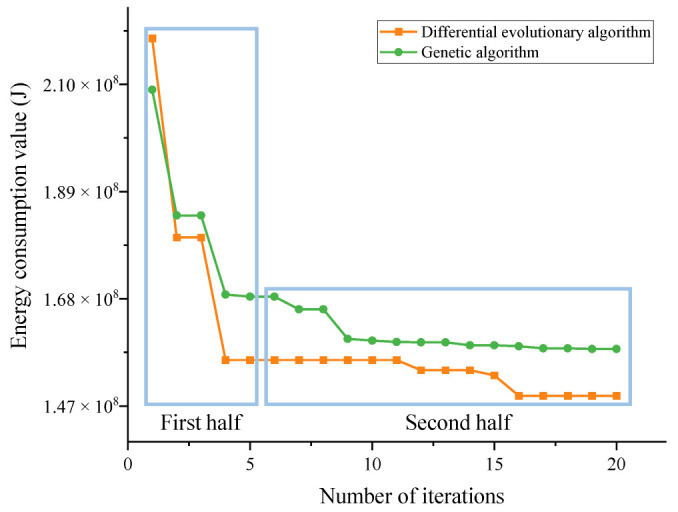
Comparison of the results of different algorithms.

Figure 8 is the optimal curve comparison diagram of genetic algorithm and differential evolution algorithm. In Figure 8, in the first half of the curve, before the fifth iteration, it can be clearly seen that the convergence rate of the differential evolution algorithm is significantly greater than that of the genetic algorithm after the third iteration. After the fourth iteration of the energy consumption value, differential evolution algorithm and genetic algorithm have pulled a certain distance. In the first half of the curve, the convergence rate of the differential evolution algorithm is faster than the genetic optimization algorithm in a short time. Additionally, the curve of the latter half of the observation, genetic algorithm in the fifth iteration to the twentieth iteration, energy consumption values still have a significant decline. The numerical decline of the differential evolution algorithm in the second half is not large. It can be seen that the differential evolution algorithm does not only converges fast at the initial time, but also quickly obtains the required global extremum, which has obvious advantages compared with the genetic algorithm.

Moreover, given its own evolutionary characteristics, it is not easy to fall into local optimum. After completing 20 iterations, the energy value of the differential evolution algorithm is lower than that of the genetic algorithm. The energy consumption of the trajectory parameters optimized by the differential evolutionary algorithm is lower than that of the trajectory parameters optimized by the genetic optimization algorithm in terms of the energy optimization of the initial trajectory parameters. Thus, the differential evolutionary algorithm has a stronger optimization capability than the genetic algorithm under this model.

### 4.2. Analysis of Trajectory Optimization Results

**Figure 9:**
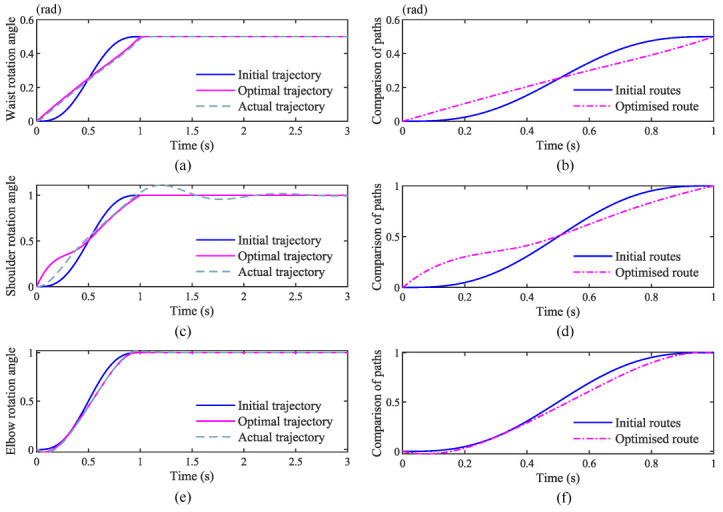
Trajectory parameter optimization. (**a**) Actual trajectory of the waist (**b**) Optimised route of the waist. (**c**) Actual trajectory of the shoulder. (**d**) Optimised route of the shoulder. (**e**) Actual trajectory of the elbow. (**f**) Optimised route of the elbow.

In Figure 9a,c,e the optimal trajectory is the trajectory parameters of the initial trajectory optimized by the planner. The actual trajectory is the trajectory profile of the optimal trajectory tracked by the proportional-derivative control algorithm. In Figure 9b,d,f the initial routes are the initial trajectory parameters collected by the bionic trajectory designed in the paper. The optimized routes are the path operation parameters with the lowest energy consumption at the waist, shoulder and elbow after optimization by the energy optimization algorithm.

In Figure 9a, proportional-derivative control algorithms can stabilize the waist joint before the system-determined stability time. The curves of the actual and optimal trajectories almost coincide during the tracking process, indicating that the proportional-derivative control algorithm can track the optimal trajectory well.

In Figure 9c, the tracking trajectory curve of the shoulder joint has some tracking error with the optimal trajectory curve. However, the tracking trajectory reaches the stable state before the stability time set by the system. In Figure 9e, the tracking trajectory of the elbow joint almost coincides with the optimal trajectory, indicating that the proportional-derivative control algorithm can achieve the tracking of the optimal trajectory.

Through the above conclusions, it can be seen that the proportional-derivative control algorithms can be used to track and control the real-time trajectory of the manipulator.

### 4.3. Analysis of Energy Optimization Results

**Figure 10:**
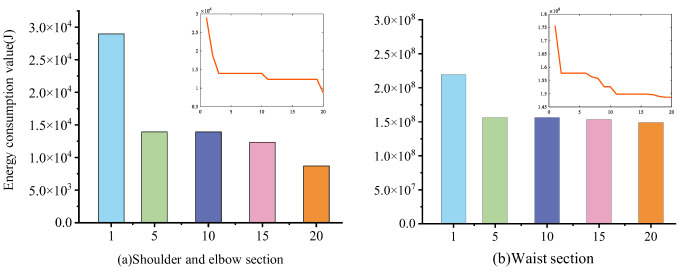
Energy consumption trends for different joints.

In Figure 10a, during optimization, the objective function based on the kinetic equations of the shoulder and elbow of the robot arm decreases continuously. The value of the objective function reaches a relatively stable situation after the 15th optimization. From the line graph, the energy consumption of the optimal trajectory has a large reduction after optimization, proving that the trajectory planner has a significant optimization effect on the objective function studied in this article.

In Figure 10b, the value of the objective function based on the waist dynamics equation of the robot arm decreases with the increase in the number of optimizations. The value of the objective function reaches a relatively stable condition after the 10th optimization. From the line graph, the energy consumption of the optimal trajectory has a large reduction after optimization. Thus, a significant reduction in waist motion energy consumption can be attributed to the trajectory planner.

To further verify the applicability of the differential evolution algorithm-based energy saving planner for various usage scenarios. Two situations that often occur in the production process are chosen to interfere with the initial trajectory parameter acquisition of the bionic demonstration system and the energy consumption model of the robotic arm.

**Figure 11:**
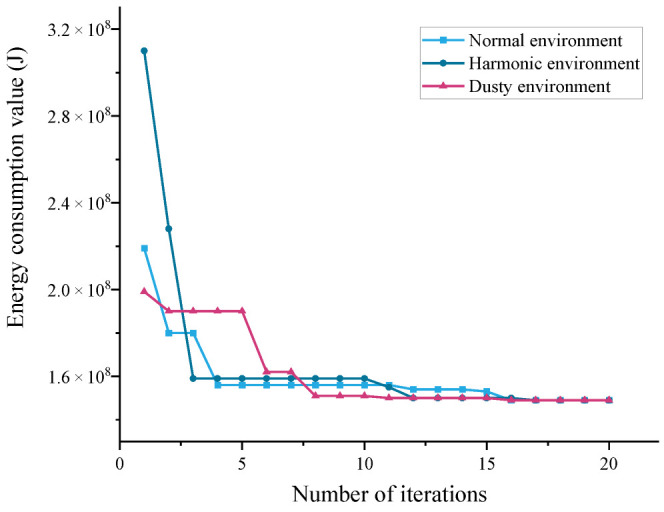
Comparison of energy optimization under different conditions.

As a disturbing factor that often occurs in industrial production, power harmonics can have a significant influence on the normal operation and energy consumption of the motor. The main cause of harmonic interference is the high-order harmonic caused by the frequency conversion of power electronic devices during the operation of the motor. Higher harmonics will affect the motor drive, especially more than three harmonics will interfere with the drive signal. High-order harmonics also have a great impact on the initial trajectory parameter acquisition of the bionic demonstration system. In Figure 11, the initial value of energy optimization changes significantly compared with the normal operation, when harmonic disturbances appear. However, with the increasing number of iterations, it can finally remain stable at the same value as in the normal case.

As power industrial robots, palletizing robots are often used in handling large materials, most of which produce large amounts of dust during handling. In the dusty environment, the viscous friction coefficient in the joint motor joint of the manipulator is mainly changed. The more serious the dust environment is, the greater the operating resistance will be, which increases the energy consumption and changes the parameters of the model. In Figure 11, the parameters of the energy consumption model change when working in dusty conditions, and the speed of energy optimization decreases compared with normal operation. However, as the number of iterations increases, it remains stable at the same value as normal. The energy optimization rate decreases compared with the normal operation; however, with the increasing number of iterations, it can still be stabilized at the same value as the normal case.

The above data show that the energy saving planner based on the differential evolutionary algorithm can complete the optimization task of the initial trajectory parameters well when it is affected by certain disturbing factors.

## 5. Conclusions

In this paper, we proposed a novel differential evolutionary algorithm-based planner to reduce the energy consumption for bionic palletizing robot. Specially, an energy saving planner is designed to optimize the bionic trajectory parameters, which is established by analyzing the dynamics of the robot arm structure. Then, a suitable initial trajectory parameter is selected to test the energy saving planner. The required objective function is established for the optimization process. Furthermore, the optimal energy saving planner based on the differential evolution algorithm is tested by simulation software, verifying that the energy consumption of the initial parameters of the bionic trajectory can be reduced after the optimization of the planner. Finally, energy consumption trajectories generated with the increase of iterations in the optimization results under different environments are analyzed. Experimental results demonstrates that the differential evolution algorithm-based planner can well reduce the energy consumption of the palletizing robot by 16%. At the same time, it is suitable for different working environments. In the future, accelerating the calculation speed of energy consumption optimization can rely on the gradual improvement of chip computing power. Moreover, the planner can collect the information of the manipulator in real time through the sensors installed on the industrial robot and construct a dynamic energy consumption model. The dynamic interaction model ensures the accuracy of the energy consumption model of the energy saving planner and can achieve better energy saving effect. Accurate energy consumption model combined with stable and efficient algorithm. This method will be an effective development direction to further improve the energy saving effect of the planner in the future.

## Figures and Tables

**Table 1:** Energy consumption reduction method.

Energy Consumption Reduction Methods						
Authors	Year	Methods	Characteristics			
			Efficiency	Stability	Accuracy	Energy Saving
Zhang et al. [6]	2022	ISSA	√	√	×	×
Hovgard et al. [7]	2021	MPT	×	×	√	√
Kyaw et al. [15]	2022	EBITR-Star	√	√	×	√
Zhu et al. [25]	2021	HSA	√	×	×	√
Zhang et al. [26]	2020	ISA	×	√	×	×
Wei et al. [27]	2019	NNS	×	√	√	√
He et al. [28]	2018	GA	×	×	√	√
Liu et al. [29]	2018	MOLA	×	√	×	√

**Table 2:** Structural parameters of palletizing robot mechanical.

Mechanical Structure	Mass (kg)	Length (m)	Distance from the Center of Mass (m)
Connecting rod 1	50.60	1.25	0.625
Connecting rod 2	30.40	0.25	0.125
Connecting rod 3	24.32	1.25	0.625
Connecting rod 4	30.40	1.50	0.500

## Data Availability

The data used to support the findings of this study are available from the corresponding author upon request.
